# Sepsis and Thrombocytopenia: A Nowadays Problem

**DOI:** 10.7759/cureus.25421

**Published:** 2022-05-27

**Authors:** Daniel A Gonzalez, Rajeswar Kumar, Saba Asif, Anoushka Bali, Ashujot Kaur Dang

**Affiliations:** 1 Medicine, Universidad Catolica Santiago de Guayaquil, Guayaquil, ECU; 2 Medicine, Rajah Muthaiah Medical College and Hospital, Chidambaram, IND; 3 Internal Medicine, Apollo Hospitals, Hyderabad, IND; 4 Research, Acharya Shri Chander College of Medical Sciences and Hospital, Jammu, IND; 5 Research, Government Medical College Patiala, Patiala, IND

**Keywords:** sirs, medical icu, severe sepsis, thrombocytopenia, sepsis

## Abstract

Sepsis is a life-threatening organ failure produced by a dysregulated host response to infection that involves 15.6% of hospital mortality. The most common signs and symptoms of sepsis are hypotension, tachypnea, fever, and leukocytosis, whether suspected or confirmed. Including a major one, thrombocytopenia is a sign that is an independent predictor of poor outcomes in patients with sepsis, increasing their mortality rate and their length of stay in the intensive care unit (ICU). So far, the ongoing treatment for this problem is securing the airway, treating hypoxemia, and providing vascular access for hydration, antibiotic delivery, and vasopressors, if needed. This article has reviewed the different possible mechanisms found for sepsis-associated thrombocytopenia, going from the most acknowledged one as decreased platelet production to the potential aftermath of sepsis itself as disseminated intravascular coagulation (DIC). This article has also discussed the future treatment for patients suffering from thrombocytopenia and sepsis, going from phase I and II trials as GI antagonists to the well-known drug aspirin as a possible treatment for this problem.

## Introduction and background

Sepsis is described as a life-threatening organ failure produced by a dysregulated host response to infection [1]. In a consensus conference, it was defined as a systemic inflammatory response syndrome (SIRS) that occurred due to an infection in 1991 by the American College of Chest Physicians and the Society of Critical Care Medicine. Then, after the 2016 consensus, SIRS was outdated for the definition of sepsis [1,2]. This pathological state affects nearly 5.9% of hospitalized patients and involves 15.6% of hospital mortality. This would mean that approximately 1.7 million US adults were hospitalized for sepsis and that 270,000 deaths occurred in 2014 [3]. Sepsis was shown to be more prevalent in males than in females (mean annual relative risk: 1.28 (95% confidence interval: 1.24-1.32)) and in non-Whites than in Caucasians (mean annual relative risk: 1.90 (95% confidence interval: 1.81-2.00)) [4]. Most of the risk factors for sepsis are related to a patient's susceptibility to infection [5]. Immunosuppressive disorders such as old and young age, acquired immune deficiency syndrome (AIDS), cancer, immunosuppressive medicines, diabetes, alcohol addiction, indwelling catheters, and other factors that influence skin integrity put patients at risk for infection [6].

The clinical features of sepsis differ considerably based on the initial location of the infection, the causative organism, the pattern of acute organ failure, the patient's underlying health state, and the time between therapy and onset [7]. Hypotension, tachycardia, tachypnea, fever, and leukocytosis are common symptoms of sepsis, whether suspected or confirmed. Signs of end-organ perfusion such as cold skin, cyanosis, and organ malfunction grow as the severity of the situation intensifies (e.g., oliguria, acute kidney injury, and altered mental status) [8,9]. A widely accepted diagnostic strategy does not exist for the clinical diagnosis of sepsis. To identify sepsis accurately, access to a varied collection of clinical data, a bedside presence, and extensive expertise with septic patients are required [10]. For sepsis patients, treatment priorities include securing the airway, treating hypoxemia, and providing vascular access for hydration and antibiotic delivery. Routine laboratory studies, serum lactate, arterial blood gases, cultures from blood, all indwelling vascular access devices, cultures from easily accessible sites such as sputum and urine, and imaging of suspected sources should all be obtained simultaneously (within 45 minutes). Still, this should not delay the administration of fluids and antibiotics [8,11]. It is critical to focus on the importance of sepsis, its high mortality, and the few resources we have to diagnose and prognosticate it. Thrombocytopenia is a major issue and an independent predictor of worse outcomes in patients with sepsis, with increased risk for increased hemorrhage, renal damage, and length of stay in the intensive care unit (ICU). Although thrombocytopenia in sepsis has not been associated as a mortality marker in numerous articles, its mere presence is a burden on the patient and the clinician managing it. This review article will emphasize the relation between sepsis and thrombocytopenia, resuming the mechanism in which sepsis tends to cause thrombocytopenia and the latest management.

## Review

Thrombocytopenia in sepsis: the proposed mechanism

The research mentioned below highlights the efforts made to identify a pathway that might explain the mechanism of thrombocytopenia in sepsis. Table 1 simplifies the classification of the mechanism.

**Table 1:** Proposed mechanism of thrombocytopenia in sepsis EDTA: ethylenediaminetetraacetic acid

Main causes
Decreased production of platelets
Interaction between platelet receptors
Immune-associated thrombocytopenia
Platelet sequestration
Consumptive coagulopathy
Other causes of thrombocytopenia in sepsis
Pseudothrombocytopenia by EDTA
Hemodilution
History of liver/spleen disease
Hematologic malignancy
Myeloproliferative disease

Decreased Production of Platelets

Firstly, it was assumed that the only mechanism of thrombocytopenia in sepsis was the decreased production of platelets, especially in patients with a critical state of sepsis, which can cause nutritional deficiencies and bone marrow failure, which can then lead to massive pancytopenia [12]. However, Segre et al. found the contrary, as a septic episode can lead to thrombopoiesis, meaning that it can increase platelet production and not the hypothesized [13]. Also, Eissa et al. studied neonatal patients with sepsis and found out that the thrombopoiesis rate is high in this population, but the platelet consumption surpasses platelet production. Therefore, they used the reticulated platelet percentage (RP%) and thrombopoietin (TPO); both markers were increased in septic patients and showed that the platelet production rate was high [14]. Moreover, according to research, significant endotoxemia in sepsis increases pro-inflammatory markers such as tumor necrosis factor-α (TNF-α), interleukin (IL)-1, IL-6, and IL-8, which are known for being thrombopoietic, meaning that it can increase platelet production [13,15]. Concluding this mechanism, Middleton et al. researched the natural history of sepsis and platelets and discovered that there are multiple changes in the expression of transcripts and translations in platelets, with the majority (64%) being upregulated [16].

Interaction Between Platelet Receptors

Toll-like receptors (TLRs), whose job is to recognize pathogen-associated molecular patterns (PAMPs) on invading microbes, serve an essential role in triggering innate immunity. Platelets are also involved in innate immunity via platelet-derived TLR4. Findings indicate that platelets express a variety of TLRs, with TLR4 playing a functional role in the control of lipopolysaccharide (LPS)-induced thrombocytopenia and TNF-α production [17,18]. Also, Stark et al. showed that platelet-derived TLR4 is sufficient to cause microvascular thrombosis in endotoxemia, irrespective of systemic TNF-α or IL-1β [19].

Platelet-receptor glycoprotein Ibα (GPIα) and the plasma protein von Willebrand factor (vWF) both limit platelet velocity in areas of vascular injury and are involved in hemostasis and thrombosis [20]. Plummer et al. discovered that several bacteria, including *Streptococcus sanguis*, have serine-rich protein A (SrpA), which is similarly recognized by GPIb and permits platelet-bacteria binding in a sialic acid-dependent way [21]. Meanwhile, Yin et al. reported that the platelet adhesion receptor GPIb-IX plays an essential role in LPS-induced thrombosis and thrombocytopenia, and they proposed that targeting GPIb as an antiplatelet approach might help manage endotoxemia [22]. Glycoprotein IIb/IIIa (GPIIb/IIIa) is the most prevalent platelet glycoprotein and plays a vital role in platelet aggregation via fibrinogen bridging. It also collaborates with fibrinogen binding, vWF, fibronectin and vitronectin, and platelet aggregation [23]. Furthermore, *Borrelia burgdorferi* had been found to bind platelets via these receptors and *Staphylococcus aureus* via fibrinogen [24,25]. Platelet-activating factor (PAF) is essential for developing LPS-induced thrombocytopenia and neutropenia in dogs. Tsuchiya et al. employed a canine model and discovered a drop in sepsis-associated thrombocytopenia (SAT) in the PAF antagonist-treated group [26]. However, the platelet-activating factor antagonist had no advantage over the placebo in terms of survival, hemodynamic state, respiratory function, or organ failure ratings in sepsis [27].

Immune-Associated Thrombocytopenia

Hemophagocytosis is an unrestrained proliferation and activation of monocytes and macrophages that aggressively eat hemopoietic cells; it has been suggested to be involved in the development of thrombocytopenia in sepsis [28]. According to Stephan et al., thrombocytopenia in some critically ill patients may be associated with the presence of platelet autoantibodies, as seen in 30% of the patients they evaluated with sepsis [29]. There is also evidence that phagocytosis may be involved in the process of SAT, as the macrophage colony-stimulating factor is overproduced when an infection is present [28]. Furthermore, a recent study used FcRIIA transgenic mice to show that this receptor plays a dominant role in immune complex-mediated thrombocytopenia and platelet sequestration in mouse models of systemic inflammation, including stimulation with immune complexes of IgG and LPS, which are highly relevant to sepsis caused by gram-negative bacteria [30].

Platelet Sequestration

Specific LPS serotypes O8 and O9 rapidly produce platelet sequestration, activation, thrombocytopenia in capillary-rich organs such as the lungs and liver, and increased mortality [31]. Triflavin, an antiplatelet peptide consisting Arg-Gly-Asp isolated from Trimeresurus Aavoviridis venom, can prevent platelet sequestration through many complex mechanisms, especially the one induced in LPS sepsis [32]. Sheu et al. suggested that triflavin either significantly prevented platelet aggregation by acting on fibrinogen and GPIIb/IIIa, resulting in decreased thromboxane A2 production, or hindered platelet adherence to subendothelial matrixes, resulting in a reversal of platelet sequestration in the blood and liver of LPS-treated rats. In the end, they reported that triflavin prevented thrombocytopenia in sepsis [33].

Consumptive Coagulopathy

Exaggerated immunological responses in sepsis are frequently associated with excessive and dysregulated activation of coagulation and thrombosis, presenting as disseminated intravascular coagulation (DIC). Microthrombi quickly grow inside small and medium arteries during DIC, causing disrupted tissue oxygenation, multiple organ failure, and circulatory collapse. The combining activation and deactivation of platelets can lead to excessive consumption, resulting in profound thrombocytopenia [34]. Severe infection is the most prevalent cause of an acute, significantly reduced platelet count (e.g., sepsis). Neame et al. claimed that moderate thrombocytopenia could be triggered by sepsis alone, but DIC is commonly the culprit when platelets fall below 50,000/L [35].

Other Causes of Thrombocytopenia During Sepsis

While the mechanisms mentioned above are the most common that can cause thrombocytopenia, a few more can be involved during sepsis. Ethylenediaminetetraacetic acid (EDTA) is frequently used as an anticoagulant in laboratory medicine. Pseudothrombocytopenia occurs during blood sample collection when EDTA-dependent antiplatelet antibodies react with GPIIb/IIIa and produce platelet agglutination and clumping in vitro, resulting in false-negative findings [36]. Hemodilution during massive crystalloid, colloid, or blood product transfusion can lead to thrombocytopenia [37]. Additionally, sepsis with a history of liver/splenic disease, hematologic malignancy, or myeloproliferative condition is frequently accompanied by significant and persistent thrombocytopenia, which is more likely due to the underlying illness than sepsis itself [38,39].

Future treatment prospects

The mere presence of thrombocytopenia is a factor in adverse prognosis in an ICU patient. At the same time, having sepsis, the patient will have a bleak outlook [2,40]. Therefore, a lot of new and old drugs focused on improving the overall outcome of patients with sepsis-associated thrombocytopenia by pinpointing the previously mentioned proposed mechanism of thrombocytopenia. Table 2 summarizes the following studies.

IL-11 acts in conjunction with IL-3, thrombopoietin, and megakaryocyte growth and development factor (MGDF) as a potent megakaryocytopoiesis and thrombopoietic factor [41]. Liu et al. performed a systematic review and meta-analysis that suggests that recombinant human interleukin-11 (rhIL-11), also known in the market as "Oprelvekin" and "Neumega," is effective and safe in the treatment of chemotherapy-induced thrombocytopenia in patients with acute leukemia. However, one of the main issues is the development of capillary leak syndrome, which occurs when fluids from the circulatory system seep into tissue outside of the circulation [42,43]. Meanwhile, Wan et al. realized in a case-control study that IL-11 has a protective impact and can hasten platelet recovery while significantly reducing the degree of inflammatory reactions, decreasing mortality in sepsis patients with thrombocytopenia [44].

Platelet-platelet binding through fibrinogen is mediated by GPIIb/IIIa receptor inhibitors; this activation leads to multiple platelets forming a lasting secondary platelet plug. In the market, the commercially available GPIIb/IIIa receptor inhibitors include abciximab, eptifibatide, and tirofiban [45,46]. Sharron et al. proved that GPIIb/IIIa inhibitors (eptifibatide) and other antiplatelet agents led to less severe sepsis and lengthened survival in a murine model of abdominal sepsis. This was found in one of their previous study, in which they reported acute sepsis-induced cytotoxic platelet expression of a granzyme B. Eptifibatide inhibits granzyme B-mediated apoptosis in the spleen and lung, which ends up slowing the progression of sepsis [47]. The "Platelet Glycoprotein IIb/IIIa in Unstable Angina: Receptor Suppression Using Integrilin Therapy (PURSUIT) Trial" described that one of the most significant adverse effects of eptifibatide was bleeding. Usually, the bleeding was mild and happened at femoral access sites, leading to increased red cell transfusions compared to placebo [48].

Thrombopoietin is an important hormone that regulates megakaryocyte formation, increasing the number of platelets [49]. Therefore, Wu et al. are now evaluating the safety and effectiveness of recombinant human thrombopoietin (rhTPO) against placebo in severe sepsis patients with thrombocytopenia. The study has an established total of 708 patients with sepsis-associated thrombocytopenia and will undergo recombinant human thrombopoietin or placebo (in a 1:1 ratio). It will have two endpoints: seven-day all-cause mortality and 28-day all-cause mortality [50]. Usually, the main concern in using thrombopoietin is the increase in blood pressure and the risk of thrombosis. However, no severe adverse reaction was reported in the studies that used rhTPO [51,52].

Anfibatide is a GPIbα antagonist that interferes with vWF and thrombin binding, consequently inhibiting the initiation of platelet adhesion and thrombosis [53]. Hou et al. conducted a clinical trial involving 94 healthy human volunteers in phase I clinical trial and showed that it could inhibit 90% of ristocetin platelet aggregation. So far, it has been concluded that anfibatide is a safe and powerful antiplatelet reagent with high promise for future antithrombotic treatment [54]. In the last phase I clinical trial using anfibatide, no serious adverse effect occurred, and no anti-anfibatide antibodies were found in the participants [53,54].

An ongoing double-blind, randomized, placebo-controlled study uses aspirin, a low-cost and widely available treatment, to safely lower sepsis-related fatalities. The overall adverse effect of aspirin is well-known, being any hemorrhagic injury such as gastrointestinal bleeding and gastric or duodenal ulcers [55]. The AspiriN to Inhibit SEPSIS trial (ANTISEPSIS) evaluates 19,000 older Australians and the use of a 100 mg dose of aspirin once daily versus placebo. The study tries to find about three possible outcomes: aspirin reduces the mortality due to sepsis, lowers the admission to ICU for sepsis, and decreases the admission to hospital for sepsis. Being an accessible and cheap drug, aspirin is a safe bet in this study with nearly zero limitations [56].

**Table 2:** Characteristics of the included studies rhIL-11: recombinant human interleukin-11, GPIIb/IIIa: glycoprotein IIb/IIIa, rhTPO: recombinant human thrombopoietin

References	Year	Design	Antiplatelet therapy	Comment
Liu et al. [42]	2020	Meta-analysis	rhIL-11	Concluded that rhIL-11 is effective for chemotherapy-induced thrombocytopenia
Sharron et al. [47]	2012	Experimental trial	GPIIb/IIIa receptor inhibitor eptifibatide	GPIIb/IIIa inhibitors and other antiplatelet agents may be beneficial in sepsis
Wu et al. [50]	2015-ongoing	Randomized, open-label, placebo-controlled	rhTPO	Thrombopoietin for severe sepsis patients with thrombocytopenia
Hou et al. [54]	2013	Clinical trial	GPIb antagonist	Anfibatide occupies around 95% of GPIb while inhibiting up to 90% of ristocetin-specific platelet aggregation
Eisen et al. [56]	2016-ongoing	Double-blinded, randomized, placebo-controlled	Low-dose aspirin	Finding out if aspirin, a low-cost and widely available treatment, lowers sepsis-related fatalities in a safe manner

## Conclusions

Any patient with new or ongoing thrombocytopenia is a poor outcome marker in the ICU; summing up, sepsis can lead to a threatening condition. So far, in every study reviewed, there is no precise proposed mechanism for thrombocytopenia in sepsis, but we are close to reaching one. This review has shared the theorized mechanisms that could be helpful in future targeting of treatment. Although many coming drugs are quite inaccessible, such as IL-11 or anfibatide and GPIbα antagonist novel drug, hopefully, other available and cheap drugs, such as aspirin, will work in the ANTISEPSIS trial. This work aims to show the relevance of finding a mechanism that genuinely fits sepsis-associated thrombocytopenia and the prospect of a treatment that may reduce this catastrophic disease.

Studies of sepsis and thrombocytopenia have increased exponentially over the last 30 years. Future clinical trials should emphasize the importance of thrombocytopenia in sepsis, understanding the latest pathway of sepsis-associated thrombocytopenia, and the therapeutics that this critical problem needs.
